# Supplementary material for: Endovascular thrombectomy in acute stroke with a large ischemic core: A systematic review and meta-analysis of randomized controlled trials
Source: PLoS Med. 2025 Apr 17;22(4):e1004484. doi: 10.1371/journal.pmed.1004484 (PMC12037071; doi:10.1371/journal.pmed.1004484)
Supplement: S1 Table — (DOCX) [file pmed.1004484.s002.docx]

| 1. Kakita H, Yoshimura S, Uchida K, Sakai N, Yamagami H, Morimoto T, et al. Impact of Endovascular Therapy in Patients With Large Ischemic Core: Subanalysis of Recovery by Endovascular Salvage for Cerebral Ultra-Acute Embolism Japan Registry 2. Stroke. 2019;50:901-908. |
| --- |
| 1. Almallouhi E, Kasab SAI, Hubbard Z, Bass EC, Porto G, Alawieh A, et al. Outcomes of Mechanical Thrombectomy for Patients With Stroke Presenting With LowAlberta Stroke Program Early Computed Tomography Score in the Early and Extended Window. JAMA Network Open. 2021;4:e2137708. |
| 1. Sarraj A, Abraham MG, Hassan AE, Blackburn S, Kasner SE, et al. Endovascular thrombectomy plus medical care versus medical care alone for large ischaemic stroke: 1-year outcomes of the SELECT2 trial. Lancet. 2024;403:731-740. |
| 1. Thomalla G, Fiehler J, Subtil F, Bonekamp S, Aamodt AH, Fuentes B, et al. Endovascular thrombectomy for acute ischaemic stroke with established large infarct (TENSION): 12-month outcomes of a multicentre, open-label, randomised trial. Lancet Neurol. 2024;23:883-892. |
| 1. Olthuis SGH, Pirson FAV, Pinckaers FME, Hinsenveld WH, Nieboer D, Ceulemans A, et al. Endovascular treatment versus no endovascular treatment after 6-24 h in patients with ischaemic stroke and collateral flow on CT angiography (MR CLEAN-LATE) in the Netherlands: a multicentre, open-label, blinded-endpoint, randomised, controlled, phase 3 trial. Lancet. 2023:22;401:1371-1380. |
