# Supplementary material for: Endovascular thrombectomy in acute stroke with a large ischemic core: A systematic review and meta-analysis of randomized controlled trials
Source: PLoS Med. 2025 Apr 17;22(4):e1004484. doi: 10.1371/journal.pmed.1004484 (PMC12037071; doi:10.1371/journal.pmed.1004484)
Supplement: S2 Table — (DOCX) [file pmed.1004484.s003.docx]

|  | RESCUE-Japan LIMIT | ANGEL-ASPECT | SELECT2 | TESLA | TENSION | LASTE |
| --- | --- | --- | --- | --- | --- | --- |
| Bias arising from the randomization process | Low risk of bias | Low risk of bias | Low risk of bias | Low risk of bias | Low risk of bias | Low risk of bias |
| Bias due to deviations from intended interventions | High risk of bias | High risk of bias | High risk of bias | High risk of bias | High risk of bias | High risk of bias |
| Bias due to missing outcome data | Low risk of bias | Low risk of bias | Low risk of bias | Low risk of bias | Low risk of bias | Low risk of bias |
| Bias in measurement of the outcome | Low risk of bias | Low risk of bias | Low risk of bias | Low risk of bias | Low risk of bias | Low risk of bias |
| Bias in selection of the reported result | Low risk of bias | Low risk of bias | Low risk of bias | Low risk of bias | Low risk of bias | Low risk of bias |
| Overall bias | High risk of bias | High risk of bias | High risk of bias | High risk of bias | High risk of bias | High risk of bias |
