# Supplementary figures and images for: Endovascular thrombectomy in acute stroke with a large ischemic core: A systematic review and meta-analysis of randomized controlled trials
Source: PLoS Med. 2025 Apr 17;22(4):e1004484. doi: 10.1371/journal.pmed.1004484 (PMC12037071; doi:10.1371/journal.pmed.1004484)

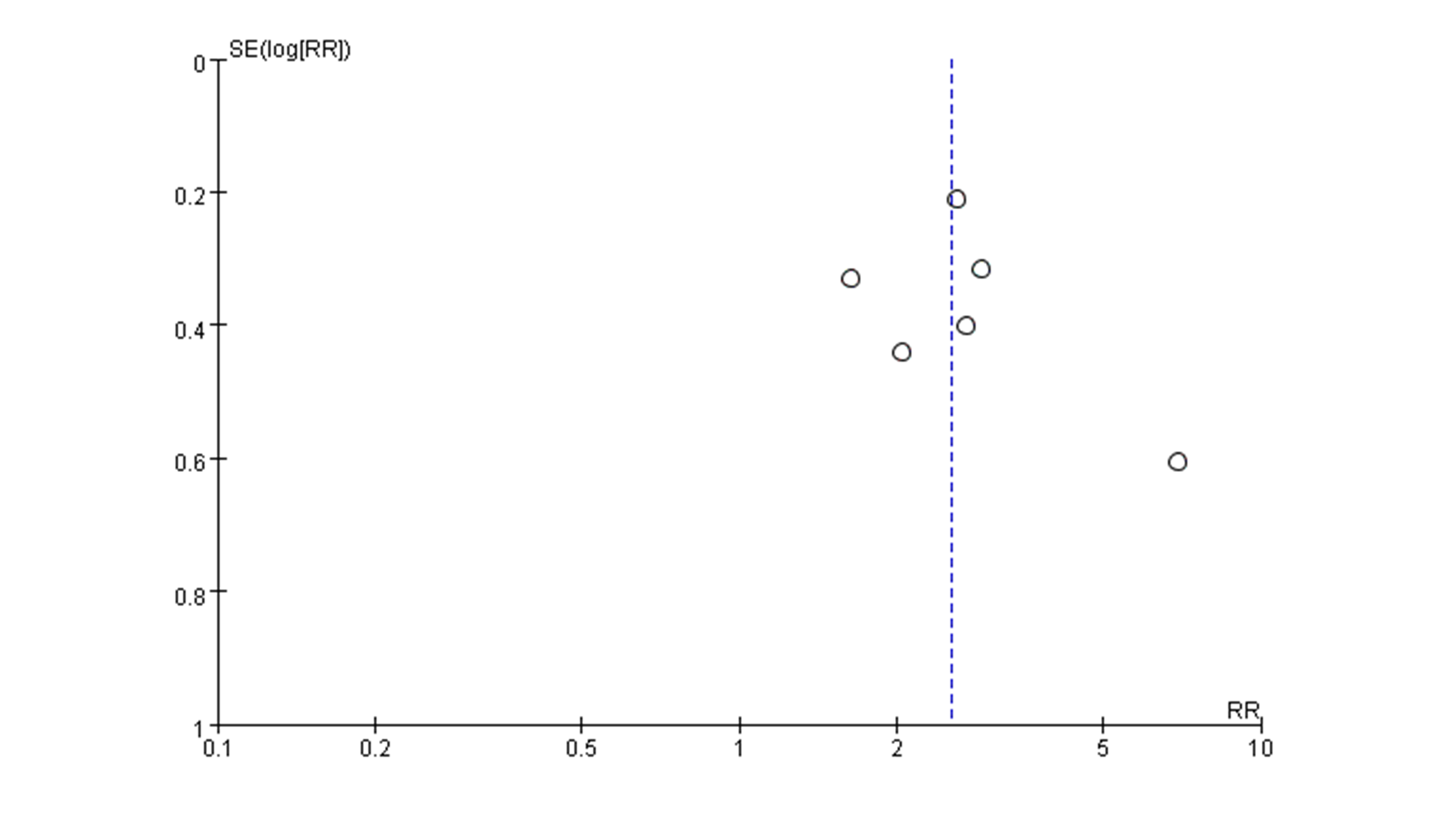

Supplement: S1 Fig — Funnel plot of included trials. RR, risk ratio; SE, standard error. (TIF) [file pmed.1004484.s004.TIF]

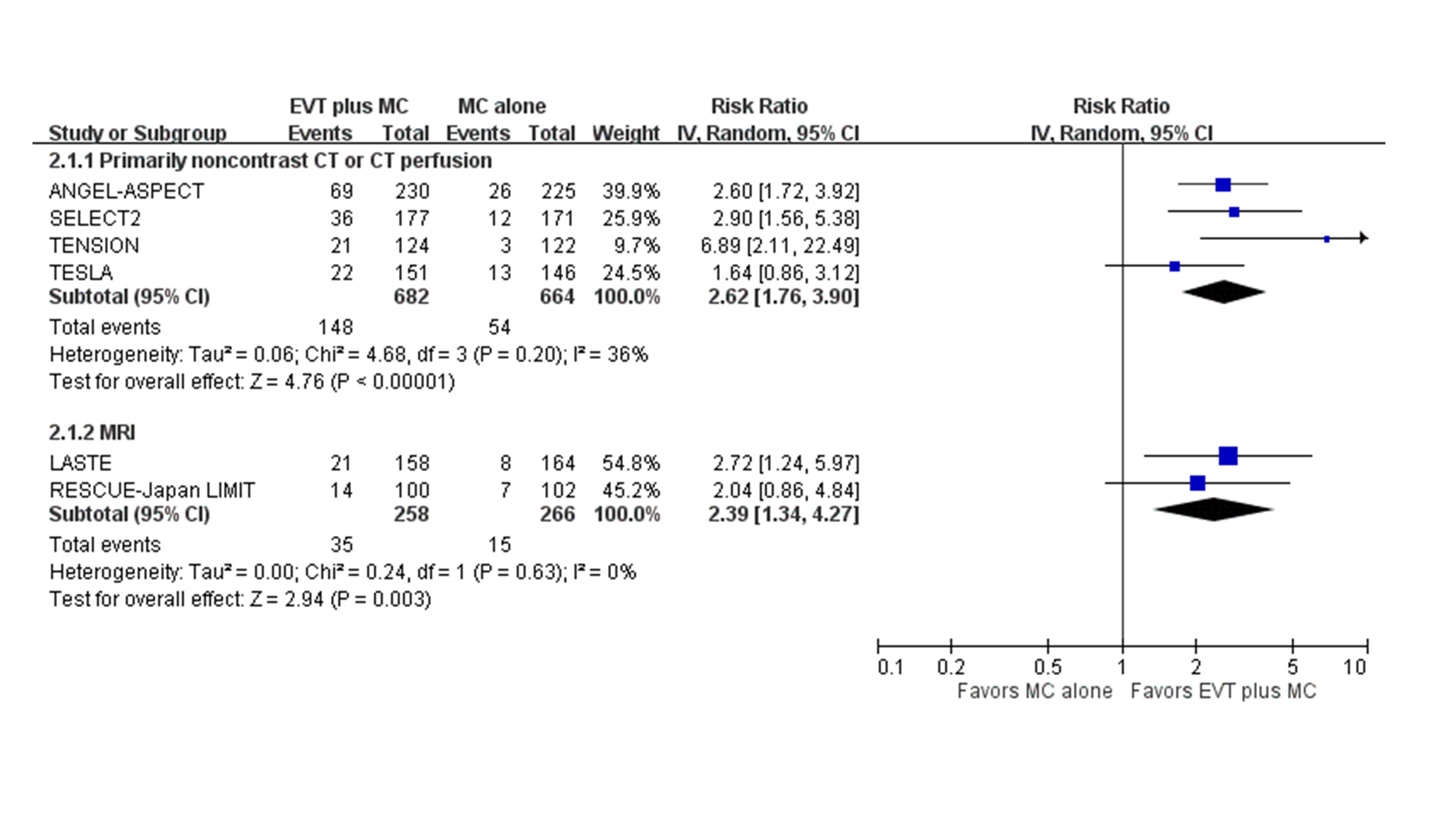

Supplement: S2 Fig — Treatment effect of EVT plus medical care compared with medical care alone on functional independence stratified by imaging modality. CT: computed tomography; EVT, endovascular thrombectomy; MRI: magnetic resonance imaging. (TIF) [file pmed.1004484.s005.TIF]

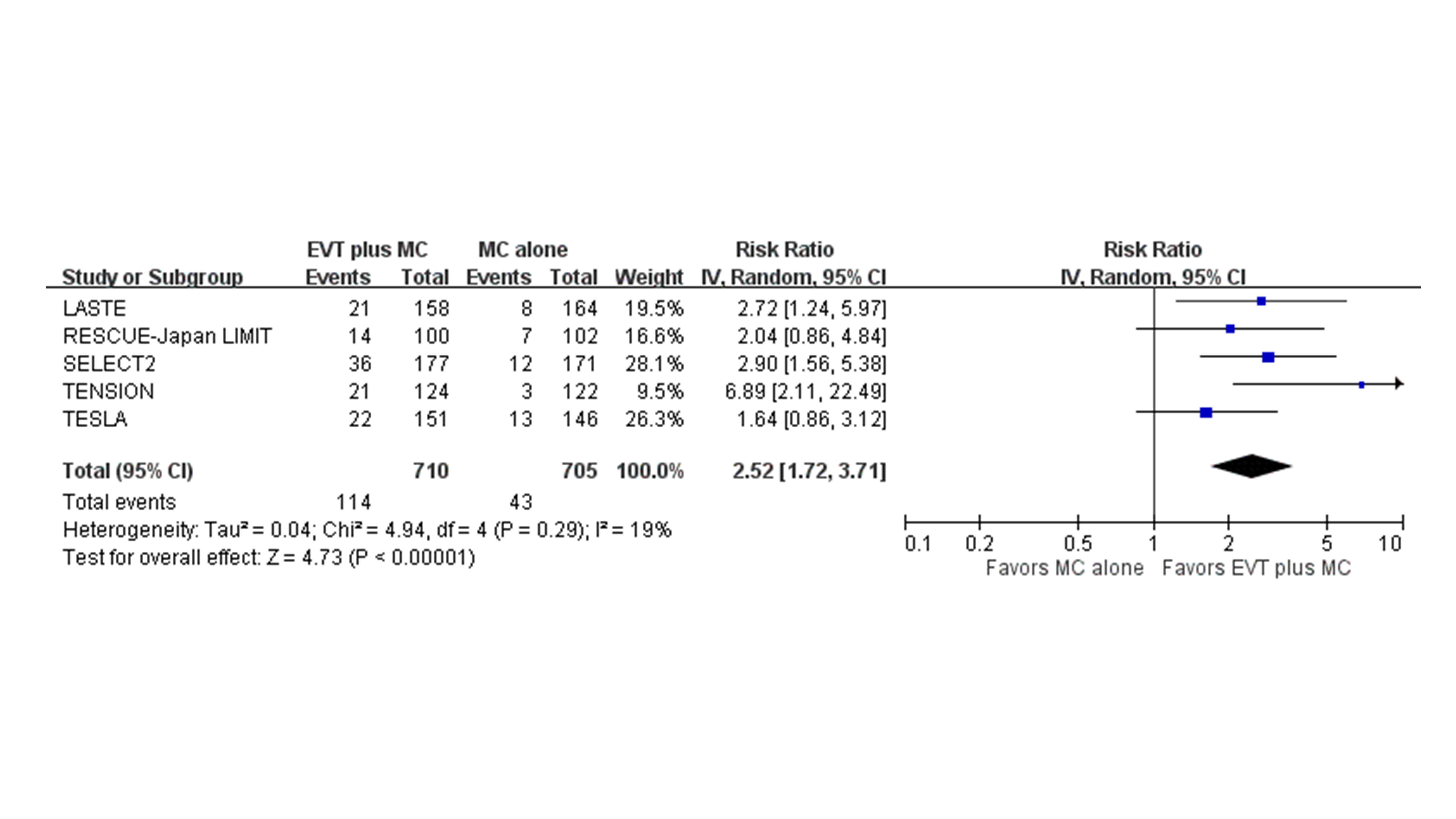

Supplement: S3 Fig — Sensitivity analysis of functional independence at 90 days, comparing EVT plus medical care with medical care alone in acute stroke with a large ischemic core due to ICA or MCA M1 occlusion, after excluding the ANGEL-ASPECT trial. CI, confidence interval; EVT, endovascular thrombectomy; ICA, internal carotid artery; IV, inverse variance; MC, medical care; MCA, middle cerebral artery; M1, M1 segment of middle cerebral artery. (TIF) [file pmed.1004484.s006.TIF]

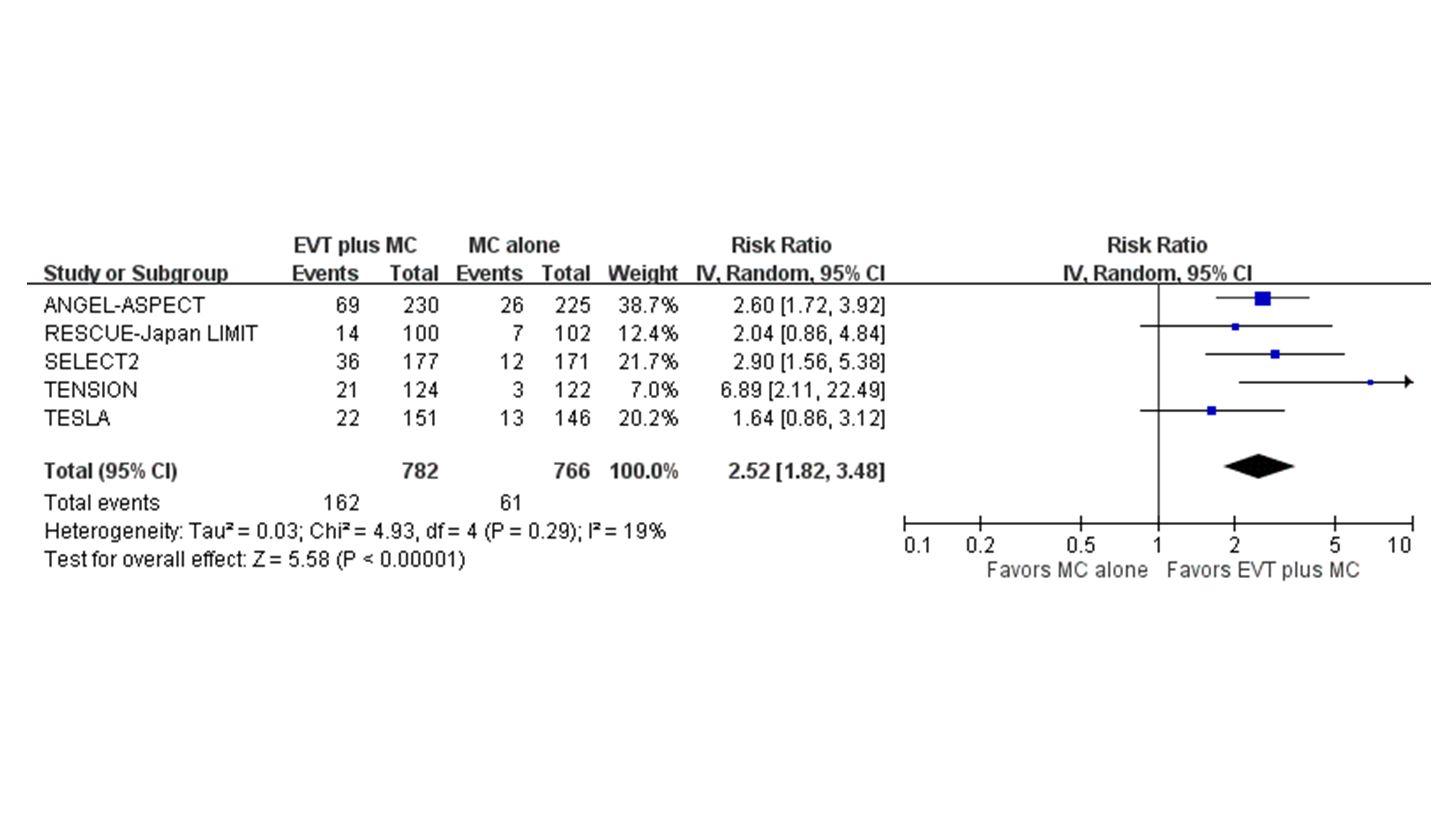

Supplement: S4 Fig — Sensitivity analysis of functional independence at 90 days, comparing EVT plus medical care with medical care alone in acute stroke with a large ischemic core due to ICA or MCA M1 occlusion, after excluding the LASTE trial. CI, confidence interval; EVT, endovascular thrombectomy; ICA, internal carotid artery; IV, inverse variance; MC, medical care; MCA, middle cerebral artery; M1, M1 segment of middle cerebral artery. (TIF) [file pmed.1004484.s007.TIF]

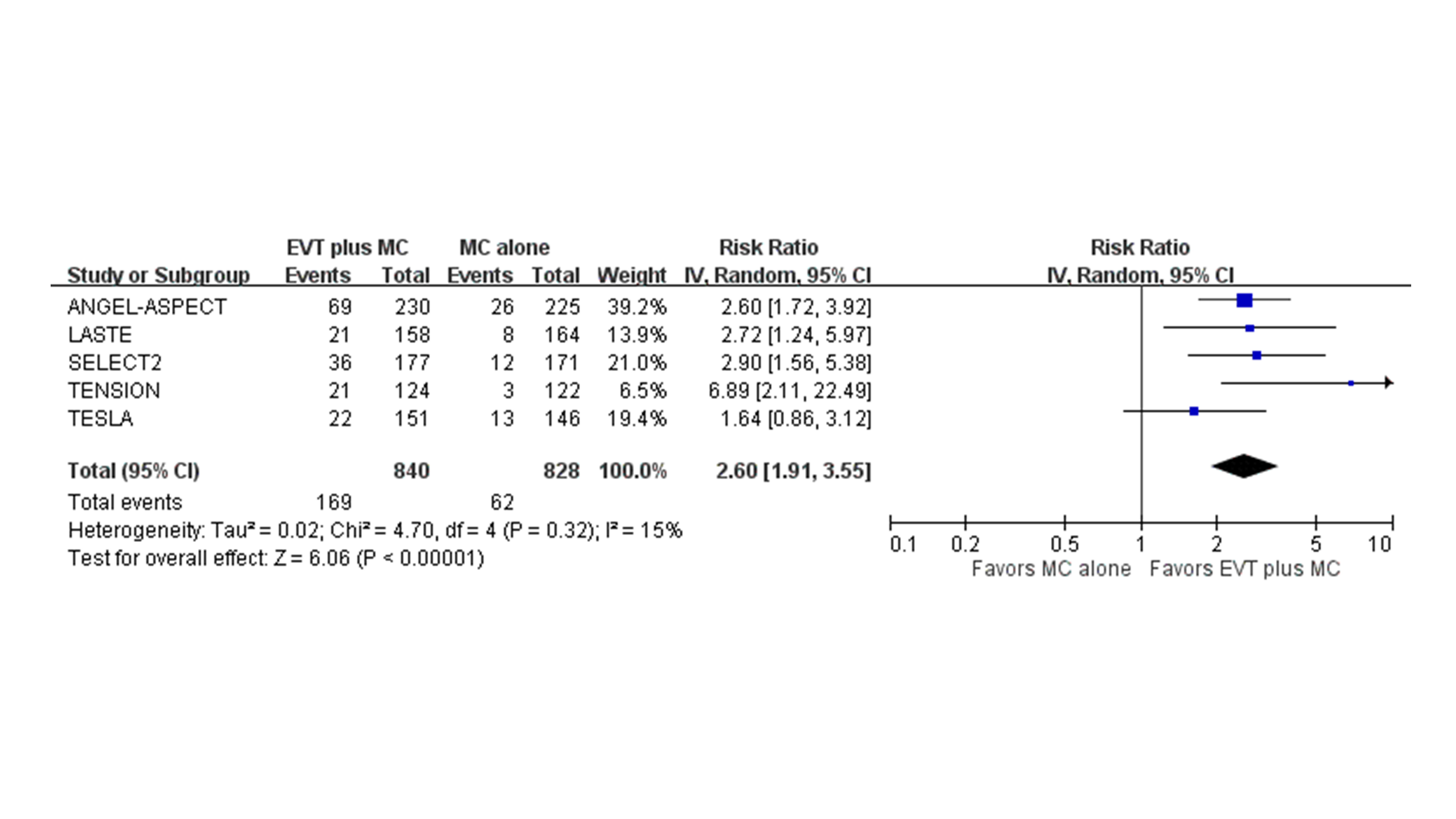

Supplement: S5 Fig — Sensitivity analysis of functional independence at 90 days, comparing EVT plus medical care with medical care alone in acute stroke with a large ischemic core due to ICA or MCA M1 occlusion, after excluding the RESCUE-Japan LIMIT. CI, confidence interval; EVT, endovascular thrombectomy; ICA, internal carotid artery; IV, inverse variance; MC, medical care; MCA, middle cerebral artery; M1, M1 segment of middle cerebral artery. (TIF) [file pmed.1004484.s008.TIF]

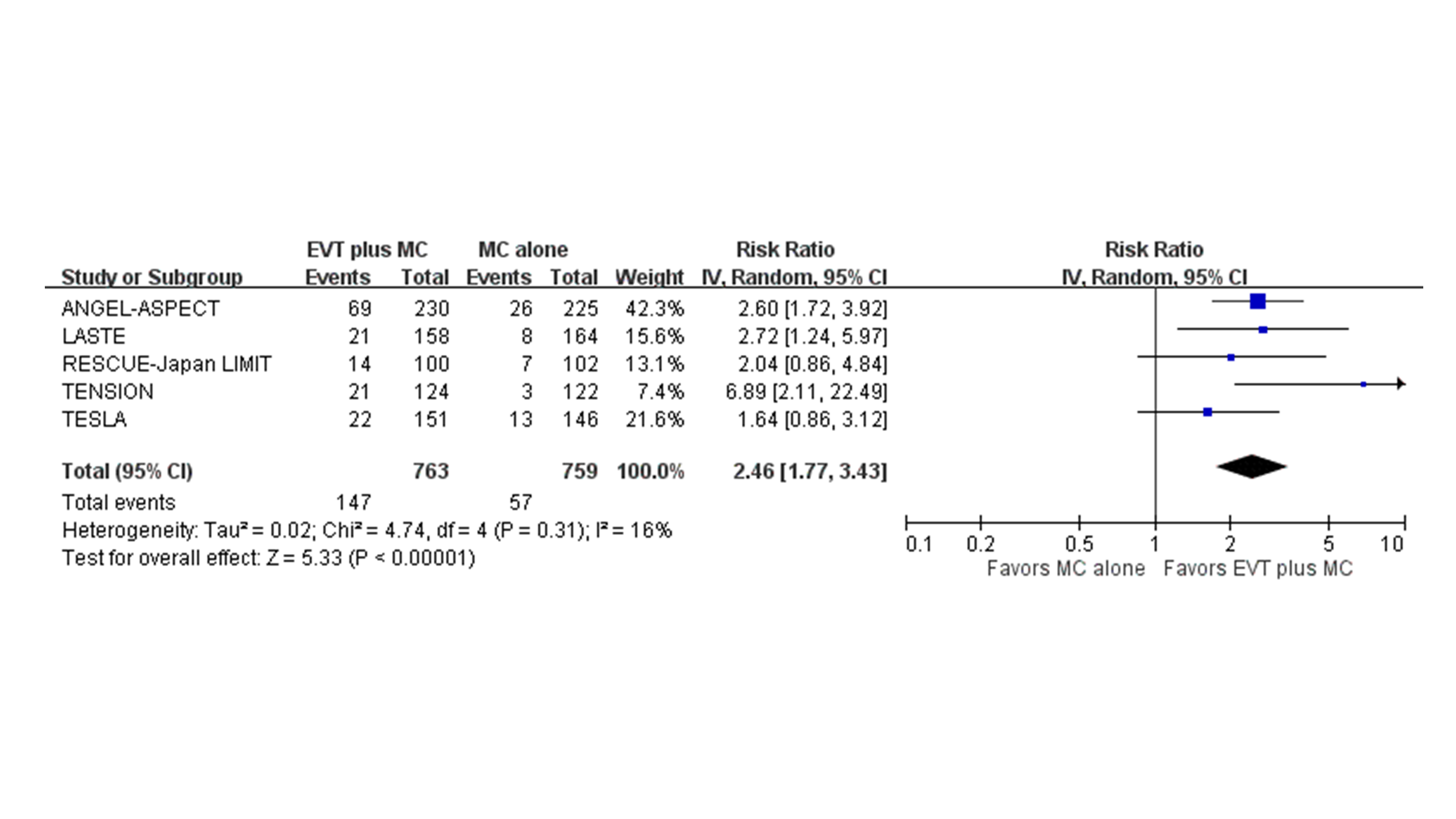

Supplement: S6 Fig — Sensitivity analysis of functional independence at 90 days, comparing EVT plus medical care with medical care alone in acute stroke with a large ischemic core due to ICA or MCA M1 occlusion, after excluding the SELECT2 trial. CI, confidence interval; EVT, endovascular thrombectomy; ICA, internal carotid artery; IV, inverse variance; MC, medical care; MCA, middle cerebral artery; M1, M1 segment of middle cerebral artery. (TIF) [file pmed.1004484.s009.TIF]

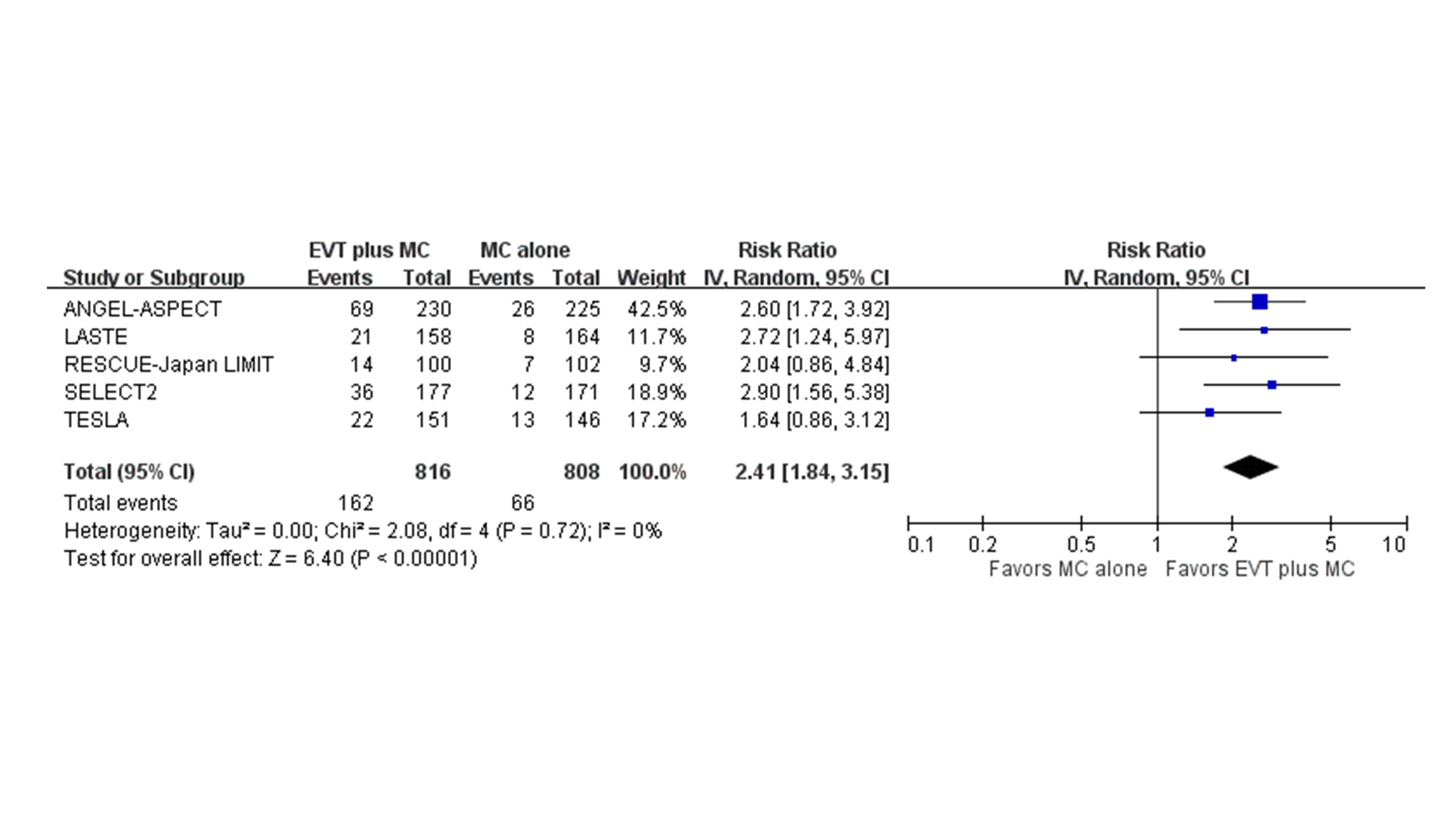

Supplement: S7 Fig — Sensitivity analysis of functional independence at 90 days, comparing EVT plus medical care with medical care alone in acute stroke with a large ischemic core due to ICA or MCA M1 occlusion, after excluding the TENSION trial. CI, confidence interval; EVT, endovascular thrombectomy; ICA, internal carotid artery; IV, inverse variance; MC, medical care; MCA, middle cerebral artery; M1, M1 segment of middle cerebral artery. (TIF) [file pmed.1004484.s010.TIF]

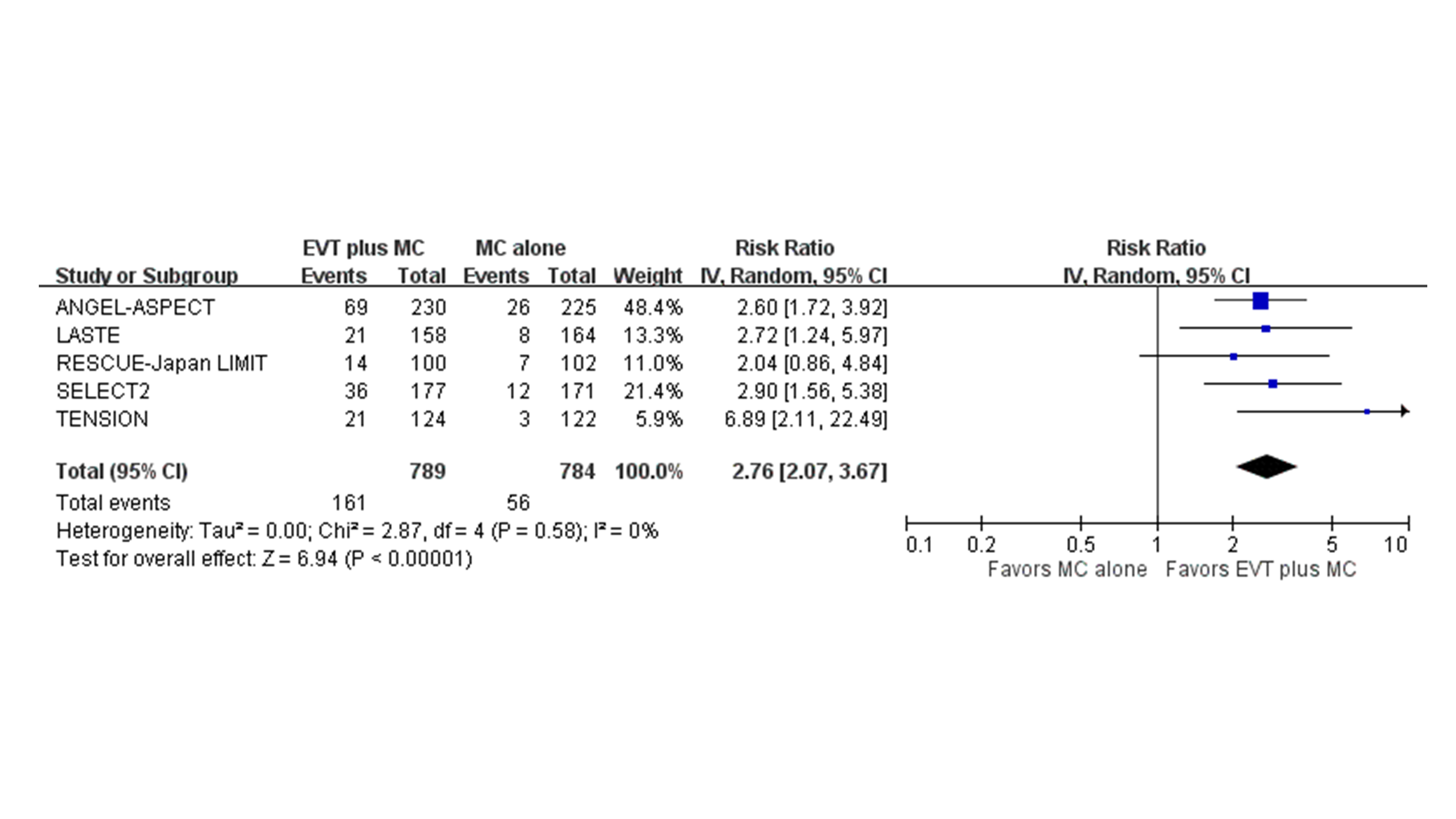

Supplement: S8 Fig — Sensitivity analysis of functional independence at 90 days, comparing EVT plus medical care with medical care alone in acute stroke with a large ischemic core due to ICA or MCA M1 occlusion, after excluding the TESLA trial. CI, confidence interval; EVT, endovascular thrombectomy; ICA, internal carotid artery; IV, inverse variance; MC, medical care; MCA, middle cerebral artery; M1, M1 segment of middle cerebral artery. (TIF) [file pmed.1004484.s011.TIF]
