# Supplementary material for: Endovascular thrombectomy in acute stroke with a large ischemic core: A systematic review and meta-analysis of randomized controlled trials
Source: PLoS Med. 2025 Apr 17;22(4):e1004484. doi: 10.1371/journal.pmed.1004484 (PMC12037071; doi:10.1371/journal.pmed.1004484)
Supplement: Attachment — Submitted filename: lin.pdf [file pmed.1004484.s015.pdf]

This paper describes a systematic review with meta–analysis of trials of endovascular thrombectomy post–stroke.

I was asked for a methodological report and I interpret that to include all aspects of the design and conduct of the study.

Points of detail

Page 3 It would perhaps be useful to have confidence intervals (CI) also for the NNT.

Page 4 If these adverse events are important enough to report here (which I believe they are) then I think they deserve the same reporting of effect size with CI as the other outcomes.

Page 6 I think the whole report follows the PRISMA guideline not just abstraction. I am not sure what validity means here. Why not just say 'We report the review following the PRISMA reporting guideline'?

Page 6 Why start the search in 2000? If the procedure was introduced then just say so.

Page 8 Typo? 'score or 5 to 6' is perhaps 'score of 5 to 6'.

Page 8 It is not clear to me what the value of these commonly quoted cut–offs for I^2 are. With the number of trials here I^2 is going to be fairly imprecisely estimated anyway so CI for it would be illuminating (Ioannidis et al., 2007).

Page 9 What funnel plots and regression tests hope to identify is small study effects. Calling it publication bias implies you know the aetiology.

Page 9 Perhaps useful to later workers wanting to repeat this to state which the 5 were which were excluded. This is always a good idea but with only 5 of them it should not be too onerous to put them in the supplement.

Page 11 My point about CI for the NNT applies here and later too.

Page 12 Just to reiterate my point about CI for I^2 my calculations indicate that the values quoted in eFigure 2 give an interval for I^2 from 0 to 78 so I would be cautious about over–interpreting the amount of heterogeneity. I used the test–based intervals from Higgins and Thompson (2002).

Page 14 Given the uncertainties involved in meta–analysis I would be more

cautious here. I assume that the authors' software estimates τ^2 using the method of moments (also known as the DerSimonian method). If I reestimate using REML, which is the default in metafor (`https://cran.r-project.org/package=metafor`), I find that for early symptomatic intracranial hemorrhage the confidence interval no longer includes the null although for death within 90 days it still does. Given that usual care is, if anything, better here I think we cannot dismiss the effect without more data

Page 15 At least for the primary outcome the results of the sensitivity analysis should be shown. They could go in the supplement.

Page 15 I doubt whether with six trials the funnel plot was ever likely to show very much but no harm including it.

Point of more substance

The authors' meta–analysis produces risk ratios so I am not clear where the absolute risk differences which underlie the NNT referred come from. Is that a separate meta–analysis? It looks plausible as running the Figure 1 data using risk difference and then taking the reciprocal does indeed give NNT 8.9 with CI 6.6 to 13.5 which agrees in central estimate to the authors' value. If this is what was done then it needs describing in the methods.

As far as I can see reference 17 consists of using experts to construct a synthetic data–set and then analysing that. If that is the case we need more details of how this was implemented both for transparency and because readers may be as unfamiliar with the method as I was. The details could, of course, go in the supplement if the authors feel they would interrupt the flow.

Summary

Mostly for clarification. As it stands if the authors gave me their data I do not think I could reproduce all of the NNT results.

Michael Dewey

References

J P T Higgins and S G Thompson. Quantifying heterogeneity in a meta–analysis. *Statistics in Medicine*, 21:1539–1558, 2002.

J Ioannidis, N Patsopoulos, and E Evangelou. Uncertainty in heterogeneity estimates in meta–analyses. *British Medical Journal*, 335:914–916, 2007.
