# Supplementary material for: Endovascular thrombectomy in acute stroke with a large ischemic core: A systematic review and meta-analysis of randomized controlled trials
Source: PLoS Med. 2025 Apr 17;22(4):e1004484. doi: 10.1371/journal.pmed.1004484 (PMC12037071; doi:10.1371/journal.pmed.1004484)
Supplement: Attachment — Submitted filename: Response to reveiwers.docx [file pmed.1004484.s017.docx]

Comments from the reviewers:

Reviewer #1: I've been tasked with reviewing a study level meta-analysis of the six major large core trials of EVT vs. medical care. I am reviewing a revised version of the manuscript that has already gone one round of peer review. Overall the study reads well and the methodology of the systematic review and meta-analysis is sound. I have no major criticisms. The limitations highlight the major drawbacks of a study level meta-analysis. Some minor comments:

1.	In the discussion, the authors state: "In EVT-treated patients in an individual participant level analysis of a subset of the completed trials, clinical outcomes worsened as ischemic injury estimates increased" - please correct, this was an individual patient level analysis of a single trial (SELECT2)

Response: Thank you for your comments. We now have revised the statement as “In EVT-treated patients of the SELECT2, clinical outcomes worsened as ischemic injury estimates increased.”

2.	In the discussion, the authors state: " A meta-analysis of individual patient level data did not find any imaging biomarker that could be used as a treatment effect modifier to decide which AIS-LVO patients should be treated with EVT or not" - please note that this IPD meta-analysis was of small/moderate core patients (HERMES).

Response: Thank you for your comments. We now have revised the statement as “A meta-analysis of individual patient-level data from acute ischemic stroke patients with small or moderate core infarcts did not find any imaging biomarker…”.

3.	One suggested additional analysis - given the heterogenity in the imaging modality used for each trial, it may be worth performing a sensivity analysis looking at the trials that primarily used Non-contrast CT or CTP (TENSION, TESLA, SELECT2, ANGLES-ASPECT) and the trials that primarily used MRI (LASTE, RESCUE-LIMIT Japan).

Response: Thank you for your comments. We now have added “Subgroup analysis of the primary outcome was conducted based on the imaging modalities used for patient selection for enrollment (non-contrast CT or CTP vs. MRI).” to the Methods section and “Subgroup analysis showed that the benefit of EVT in achieving functional independence was not significantly different between patients selected by non-contrast CT or CTP versus those selected by MRI (RR 2.62, 95% CI [1.76, 3.91] vs. RR 2.39, 95% CI [1.34, 4.27]; p=0.80 for interaction) (S8 Fig).” to the Results section and S8 Fig.

Reviewer #2

This paper describes a systematic review with meta–analysis of trials of en- dovascular thrombectomy post–stroke.

I was asked for a methodological report and I interpret that to include all aspects of the design and conduct of the study.

Points of detail

4. Page 3 It would perhaps be useful to have confidence intervals (CI) also for the NNT.

Response: Thank you for your comments. We now have added NNT 9, 95% CI [6, 15]) for greater funtional independence and NNT 7, 95% CI [6, 11] for a lower risk of requiring constant care or death.

5. Page 4 If these adverse events are important enough to report here (which I believe they are) then I think they deserve the same reporting of effect size with CI as the other outcomes.

Response: Thank you for your comments. We now have revised the statement as “EVT compared with medical care alone was associated with a trend toward higher proportion of patients with early symptomatic intracranial hemorrhage (RR 1.65, 95% CI [1.00, 2.70]; p=0.05). The rates of death were not significantly different between the EVT and medical care alone groups (RR 0.86, 95% CI [0.72, 1.02]; p=0.08).” to the Abstract section.

6. Page 6 I think the whole report follows the PRISMA guideline not just abstraction. I am not sure what validity means here. Why not just say ‘We report the review following the PRISMA reporting guideline’?

Response: Thank you for your comments. We now have adjusted statement as “This study is reported as per the Preferred Reporting Items for Systematic Reviews and Meta-Analyses (PRISMA) guideline (S1 Checklist).” as your and editor’s suggestion.

7. Page 6 Why start the search in 2000? If the procedure was introduced then just say so.

Response: Thank you for your comments. We now have revised the statement as “… for studies published from January 1, 2000 (the first modern thrombectomy device was induced in early 2000s), to September 25, 2024…” to the Methods.

8. Page 8 Typo? ‘score or 5 to 6’ is perhaps ‘score of 5 to 6’.

Response: Thank you for your comments. We now have corrected the typo, change “or” to “of”.

9. Page 8 It is not clear to me what the value of these commonly quoted cut–offs for I2 are. With the number of trials here I2 is going to be fairly imprecisely estimated anyway so CI for it would be illuminating (Ioannidis et al., 2007).

Response: Thank you for your comments. The points were well-taken and we agreed that with the number of trials here I^2^ is going to be fairly imprecisely estimated anyway. However, we think even when we calculate the CI of I^2^, it might be difficult for us to interpretate the clinical meaning of the (likely) very wide range of CI of I^2^ of each outcome. Therefore we decided not calculating and presneting I^2^ and its CI to assess the heterogeniety. We now have deleted the quoted cutoffs of I^2^ and its referecens in the Methods and Results sections and revised the statement as “All statistical tests for heterogeneity are weak, including I^2^. (Ioannidis et al., 2007). Given the small number of trials in our study, I^2^ is going to be fairly imprecisely estimated anyway so CI for it might be illuminating. (Ioannidis et al., 2007). However, interpreting the clinical significance of wide CI of I² remains challenging. Therefore, we assessed heterogeneity primarily through visual inspection of forest plots for each outcome.” to the Methods section.

Also, we now have added the following statement to the Results section: “Heterogeneity might exist among studies, especially between the TENSION trial and the TESLA trial, by visually inspecting the forest plots.” for the primary outcome, “Heterogeneity might exist among the the 2 trials (the RESCUE-Japan LIMIT and the TENSION trial) vs. the other 4 trials, by visually inspecting the forest plots.” for the mRS shift outcome, “Heterogeneity might exist among the the 2 trials (the ANGEL-ASPECT trial and the TESLA trial) vs. the other 4 trials, by visually inspecting the forest plots.” for ambulation outcome, “Heterogeneity might exist among studies by visually inspecting the forest plots.” for being nondiabled and early neurologic improvement outcomes, “Heterogeneity might exist among studies, especially between the RESCUE-Japan LIMIT and the TESLA trial, by visually inspecting the forest plots” for requiring constant caring or death outcome, “Heterogeneity might exist among the the 2 trials (the ANGEL-ASPECT trial and the TESLA trial) vs. the LASTE trial, by visually inspecting the forest plots.” for death outcome, and “Heterogeneity might exist among the the SELECT2 trial vs. the other 5 trials, by visually inspecting the forest plots.” for early symptomatic intracranial hemorrhage outcome.

10. Page 9 What funnel plots and regression tests hope to identify is small study effects. Calling it publication bias implies you know the aetiology.

Response: Thank you for your comments. The point was well-taken. We now have revised it as “Potential small study effects were assessed visually by a funnel plot displaying standard error as the measure of sample size and risk ratio as the measure of treatment effect.” to the Methods section.

11. Page 9 Perhaps useful to later workers wanting to repeat this to state which the 5 were which were excluded. This is always a good idea but with only 5 of them it should not be too onerous to put them in the supplement.

Response: Thank you for your comments. We now put this 5 studies in S1 Table.

**S1 Table. Title: Studies excluded after detailed assessment**

1. Kakita H, Yoshimura S, Uchida K, Sakai N, Yamagami H, Morimoto T, et al. Impact of Endovascular Therapy in Patients With Large Ischemic Core: Subanalysis of Recovery by Endovascular Salvage for Cerebral Ultra-Acute Embolism Japan Registry 2. Stroke. 2019;50:901-908.

2. Almallouhi E, Kasab SAI, Hubbard Z, Bass EC, Porto G, Alawieh A, et al. Outcomes of Mechanical Thrombectomy for Patients With Stroke Presenting With LowAlberta Stroke Program Early Computed Tomography Score in the Early and Extended Window. JAMA Network Open. 2021;4:e2137708.

3. Sarraj A, Abraham MG, Hassan AE, Blackburn S, Kasner SE, et al. Endovascular thrombectomy plus medical care versus medical care alone for large ischaemic stroke: 1-year outcomes of the SELECT2 trial. Lancet. 2024;403:731-740.

4. Thomalla G, Fiehler J, Subtil F, Bonekamp S, Aamodt AH, Fuentes B, et al. Endovascular thrombectomy for acute ischaemic stroke with established large infarct (TENSION): 12-month outcomes of a multicentre, open-label, randomised trial. Lancet Neurol. 2024;23:883-892.

5. Olthuis SGH, Pirson FAV, Pinckaers FME, Hinsenveld WH, Nieboer D, Ceulemans A, et al. Endovascular treatment versus no endovascular treatment after 6-24 h in patients with ischaemic stroke and collateral flow on CT angiography (MR CLEAN-LATE) in the Netherlands: a multicentre, open-label, blinded-endpoint, randomised, controlled, phase 3 trial. Lancet. 2023;401:1371-1380.

12. Page 11 My point about CI for the NNT applies here and later too.

Response: Thank you for your comments. The point was well-taken. We now have added CI for all the NNT in the text and Abstract.

13. Page 12 Just to reiterate my point about CI for I2 my calculations indicate that the values quoted in eFigure 2 give an interval for I2 from 0 to 78 so I would be cautious about over–interpreting the amount of heterogeneity. I used the test–based intervals from Higgins and Thompson (2002).

Response: Thank you for your comments. The point was well-taken. We now have revised the text as “Heterogeneity might exist among the the 2 trials (the RESCUE-Japan LIMIT and the TENSION trial) vs. the other 4 trials, by visually inspecting the forest plots.” to the Results section.

14. Page 14 Given the uncertainties involved in meta–analysis I would be more

cautious here. I assume that the authors’ software estimates τ 2 using the method of moments (also known as the DerSimonian method). If I reestimate using REML, which is the default in metafor (https:// cran.r-project.org/package=metafor), I find that for early symptomatic intracranial hemorrhage the confidence interval no longer includes the null although for death within 90 days it still does. Given that usual care is, if anything, better here I think we cannot dismiss the effect without more data.

Response: Thank you for your comments. The point was well-taken. We now have revised the statement as “…EVT compared with medical care alone was associated with a trend toward higher proportion of patients with early symptomatic intracranial hemorrhage…”. We also revised the statement as “EVT compared with medical care alone was associated with a trend toward higher proportion of patients with early symptomatic intracranial hemorrhage in AIS-LVO patients with large ischemic cores (6 trials; absolute risk, 4.4% vs. 2.7%; RR 1.65, 95% CI [1.00, 2.70]; p=0.05) (S7 Fig)” in the Abstract

15. Page 15 At least for the primary outcome the results of the sensitivity analysis should be shown. They could go in the supplement.

Response: Thank you for your comments. The point was well-taken. We now have added “Sensitivity test excluding individual trials serially yielded pooled results similar to the overall pooled estimates of the primary outcome, indicating that no single study had a disproportionate influence on the overall effect size (S9-14 Fig).” to the Results section. Also, we have added “Sensivity tests excluding individual trials serially for efficacy outcomes and safety outcomes are presented in Table 2 and Table 3.” to the Reesults section.

**Table 2. Title: Sensitivity tests for efficacy outcomes**

|  | Functional independence, RR (95% CI), 6 trials | Reduced disability, OR (95% CI), 6 trials | Ambulation, RR (95% CI), 6 trials | Being nondisabled, RR (95% CI), 6 trials | Early neurologic improvement, RR (95% CI), 5 trials |
| --- | --- | --- | --- | --- | --- |
| All trials pooled | 2.53 (1.95-3.29) | 1.63 (1.38-1.93) | 1.92 (1.50-2.44) | 2.64 (1.71-4.08) | 2.35 (1.79-3.10) |
| ANGEL-ASPECT excluded | 2.52 (1.72-3.71) | 1.73 (1.42-2.11) | 2.10 (1.69-2.59) | 2.33 (1.37-3.96) | 2.31(1.68-3.16) |
| LASTE excluded | 2.52 (1.82-3.48) | 1.66 (1.33-2.08) | 1.77 (1.41-2.23) | 2.67 (1.68-4.23) | 2.24 (1.55-3.23) |
| RESCUE-Japan LIMIT excluded | 2.60 (1.91-3.55) | 1.56 (1.33-1.82) | 1.86 (1.43-2.42) | 2.77 (1.75-4.38) | 2.18 (1.62-2.94) |
| SELECT2 excluded | 2.46 (1.77-3.43) | 1.70 (1.36-2.13) | 2.02 (1.40-2.91) | 2.54 (1.60-4.04) | 2.57 (1.90-3.48) |
| TENSION excluded | 2.41 (1.84-3.15) | 1.53 (1.34-1.75) | 1.86 (1.43-2.42) | 2.49 (1.58-3.93) | NA |
| TESLA excluded | 2.76 (2.07-3.67) | 1.68 (1.38-2.04) | 2.04 (1.53-2.72) | 3.11 (1.87-5.18) | 2.53 (1.77-3.60) |

Legends: Sensitivity tests excluding individual trials seriallyo for efficacy outcomes, comparing EVT plus medical care versus medical care alone in AIS-LVO patients with large ischemic cores

AIS-LVO, acute ischemic stroke due to large-vessel occlusions; CI: confidence interval; EVT, endovascular thrombectomy; NA: not applicable; OR: odds ratio; RR: risk ratio

**Table 3. Title: Sensitivity tests for safety outcomes**

|  | Requiring constant care or death, RR (95% CI), 6 trials | Death, RR (95% CI), 6 trials | Early symptomatic intracranial hemorrhage, RR (95% CI), 6 trials |
| --- | --- | --- | --- |
| All trials pooled | 0.74 (0.66-0.84) | 0.86 (0.72-1.02) | 1.65 (1.00-2.70) |
| ANGEL-ASPECT excluded | 0.73 (0.64-0.83) | 0.82 (0.69-0.99) | 1.45 (0.81-2.60) |
| LASTE excluded | 0.75 (0.65-0.87) | 0.92 (0.80-1.06) | 1.73 (1.01-2.95) |
| RESCUE-Japan LIMIT excluded | 0.77 (0.69-0.85) | 0.87 (0.72-1.05) | 1.60 (0.91-2.79) |
| SELECT2 excluded | 0.73 (0.64-0.85) | 0.84 (0.68-1.04) | 1.74 (1.05-2.88) |
| TENSION excluded | 0.76 (0.67-0.87) | 0.88 (0.71-1.09) | 1.82 (1.04-3.19) |
| TESLA excluded | 0.72 (0.64-0.80) | 0.82 (0.69-0.98) | 1.54 (0.92-2.60) |

Legends: Sensitivity tests excluding individual trials seriallyo for safety outcomes, comparing EVT plus medical care versus medical care alone in AIS-LVO patients with large ischemic cores

AIS-LVO, acute ischemic stroke due to large-vessel occlusions; CI: confidence interval; EVT, endovascular thrombectomy; NA: not applicable; RR: risk ratio

16. Page 15 I doubt whether with six trials the funnel plot was ever likely to show very much but no harm including it.

Response: Thank you for your comments. We now avoid to use the term “publication bias” and revised the statement as “The funnel plot did not show major asymmetry (S15 Fig).”

Point of more substance

17. The authors’ meta–analysis produces risk ratios so I am not clear where the absolute risk differences which underlie the NNT referred come from. Is that a separate meta–analysis? It looks plausible as running the Figure 1 data using risk difference and then taking the reciprocal does indeed give NNT 8.9 with CI 6.6 to 13.5 which agrees in central estimate to the authors’ value. If this is what was done then it needs describing in the methods.

Response: Thank you for your comments. The point was well-taken. We now have added: To aid interpretation of the results of a meta-analysis of risk ratios, we computed an absolute risk reduction or number needed to treat (NNT). In order to do this, an assumed comparator risk (ACR) (risk that the outcome of interest would occur with the comparator intervention) was required. The computation proceeds as follows: (ref 16: Schünemann HJ, Vist GE, Higgins JPT, Santesso N, Deeks JJ, Glasziou P, et al. 15.4.4.2 Computing risk differences or NNT from a risk ratio. In: Higgins JPT TJ, Chandler J, Cumpston M, Li T, Page MJ, Welch VA, editors. Cochrane Handbook for Systematic Reviews of Interventions Version 6.5, 2024. Cochrane. Available from <https://training.cochrane.org/handbook/archive/v6/chapter-15>

18. As far as I can see reference 17 consists of using experts to construct a synthetic data–set and then analysing that. If that is the case we need more details of how this was implemented both for transparency and because readers may be as unfamiliar with the method as I was. The details could, of course, go in the supplement if the authors feel they would interrupt the flow.

Response: We are grateful for this comment and have revised the manuscript to address it. Of the three different methods to derive an NNT for an ordinal outcome used in original reference 17 (now ref 18), we actually employed the algorithmic method, not the expert method. We made this choice because the algorithmic method avoids concern for potential subjective influences associated with the expert method. As the algorithmic method is likely also to be unfamiliar to readers, we have added this passage describing it to the Methods section:

      "The min-max-mean algorithmic method was used to derive the NNT for the ordinal outcome, conducted according to prior described methodology. (now ref 18) The maximum possible NNT compatible with the data was derived by completing the joint outcome table following the rule that every patient who benefits from therapy does so by improving only the minimum possible number of levels compatible with the final trial group outcome distributions. The minimum possible NNT compatible with the data was derived by completing the table following the rule that every patient who benefits from therapy does so by improving the maximum possible number of grades compatible with the group data. The central value within the possible range was obtained by calculating the geometric mean of the minimum and maximum NNTs."

We also added “NNT, 3 [minimum possible NNT 2, maximum possible NNT 6]).” for mRS shift analysis outcome to the Results section of text and Abstract.

19. Summary

Mostly for clarification. As it stands if the authors gave me their data I do not think I could reproduce all of the NNT results.

Michael Dewey

Response: Thank you for your comments. We now have added “Data Availability: All the data used to calculate the primary, secondary, and safety outcomes are included in S1 Data and S2 Data.”

References

J P T Higgins and S G Thompson. Quantifying heterogeneity in a meta– analysis. Statistics in Medicine, 21:1539–1558, 2002.

J Ioannidis, N Patsopoulos, and E Evangelou. Uncertainty in heterogeneity estimates in meta–analyses. British Medical Journal, 335:914–916, 2007.

Reviewer #3: The authors present a meta-analysis of the 6 randomized controlled trials comparing medical management to endovascular therapy for large vessel occlusion acute ischemic stroke in patients with large ischemic core. Pooled results are reported to show at 90 days, EVT (+MM) vs MM alone was associated with greater functional independence, defined as mRS 0-2 (RR 2.53, NNT 9), and reduced disability (common OR 1.63, NNT 4). EVT had a lower risk of requiring constant care or death (NNT 7). Rates of death and early sICH did not significantly differ between EVT vs MM alone. The authors conclude EVT may be associated with improved functional outcomes and reduced severe disability or death at 90d for acute LVO stroke with large ischemic core.

20. A meta-analysis evaluating the same 6 trials by Chen et al was already published in JNIS earlier this year. There are some statistics provided here that were not reported in the Chen paper (ex NNT for various outcomes), but the existence of a pre-existing meta-analysis of the exact same trials (and what this article provides in terms of novelty) is not acknowledged/provided. I would recommend doing this.

Response: Thank you for your comments. The point was well-taken. We now have added a paragraph “The current study was distinct from the study by Chen et al [35] in several aspects. First, Chen et al. published their meta-analysis of 6 randomized trials in January 2024, predating the publication of the LASTE trial (May 2024) [8] and the online publication of the TESLA trial (September 2024) [10]. Although Chen et al [35] cited the LASTE trial protocol [36], this protocol contained no outcome data. Therefore, the accuracy of the data from these two trials in Chen's meta-analysis cannot be verified [35]. Second, given that these trials included patients with large ischemic cores, it was essential to determine whether EVT reduced the risk of the very poor outcome (mRS 5 to 6) compared with medical care alone. Unlike Chen's analysis [35]. our meta-analysis demonstrated that EVT significantly reduced the very poor outcome compared with medical care.” to the Discussion section.

21. The authors' conclusion, as stated in the abstract, does not contain any caveats aside from the word "may". In their discussion, the authors acknowledge that the outcomes in the trials analyzed are not equivalent to patients treated with small/moderate sized cores in prior trials (which is not surprising). It also acknowledges some (but not all) of the limitations of the large core trials included in the meta-analysis. However, their conclusion--which is often the only thing many practitioners read—does not acknowledge any of these limitations and may be interpreted by readers only skimming the abstract to imply any patient population would benefit similarly from EVT as those with small/moderate core infarcts. The conclusion is incomplete and merits editing to account for the many limitations the data from these trials provide (Ex--should a 95 year old patient with a baseline mRS 3, an ASPECTS of 2, a dominant M2 occlusion, a LSW 16 hours prior undergo EVT based on the results of this meta-analysis? The conclusion provided implies yes, whereas I and I'd venture to guess most physicians familiar with these trials would argue absolutely not).

Response: Thank you for your comments. The point was well-taken. We now have revised the Conclusion of the Abstract as “Among ischemic stroke patients with larger moderate core or large core due to ICA or MCA M1 occlusion who were nondisabled before stroke, EVT combined with medical care compared with medical care alone may be associated with improved functional outcomes and reduced severe disability or death at 90 days.” We also revised the relevant phrases in the Results and Conclusions of the text.

22. While the CI includes 1.0 for early sICH, it certainly trended toward being higher in the EVT group and probably merits a mention of this.

Response: Thank you for your comments. The point was well-taken. We now have revised the statement as “EVT compared with medical care alone was associated with a trend toward higher proportion of patients with early symptomatic intracranial hemorrhag” to the Results section of the text and Abstract.

Reviewer #4: This manuscript presents a comprehensive meta-analysis on endovascular therapy (EVT) for large ischemic core strokes, a topic of great clinical importance. The adherence to PRISMA guidelines, and robust statistical methods enhances the reliability of the findings.

23. The manuscript is well-organized, with clear delineation of methods, results, and discussion. Phrasing "940 been randomly assigned" should be corrected to "940 were randomly assigned."

Response: Thank you for your comments. We now have corrected the phrase to "940 were randomly assigned."

24. The authors conclude that EVT may be justified despite diminished benefits in patients with large cores. As this is supported by the data, it may warrant discussion of ethical considerations, such as resource allocation.

Response: Thank you for your comments. We now have added “The findings of this study provide support for the ethics of pursuing EVT in patients with large ischemic cores. The magnitude of benefit on the primary functional independence (mRS 0-2) outcome was substantial – an absolute increase of 12%, yielding a NNT of 9. While not as large as the extraordinary benefit of EVT for small core patients, this effect magnitude on functional independence actually exceeds that of thrombolytic therapy administered within 3 hours of onset (absolute increase 8.5%, NNT 11) (Mun et el. Stroke 2022). It also well exceeds the 3.1-5% absolute increase judged by neurologists and neurointerventionalists as the minimal clinically important difference for adopting into clinical practice a new population for endovascular thrombectomy (Lin and Saver. Front Neurology 2020). Given the significant clinical benefit demonstrated, allocating resources for EVT in large-core stroke patients is evidence-based and clinically justified.” to the Discussion.

25. Finally, the authors deserve commendation for highlighting the critical issue of initial CT perfusion in the early hours overestimating the final infarct core. Relying on the CT perfusion mismatch concept to select patients for endovascular treatment (EVT) will inadvertently exclude individuals who could still benefit from reperfusion. This is an important factor to consider and may significantly impact analysis. Also, it is crucial to finally move beyond just the quantity of infarcted tissue and consider the infarct's location and eloquent areas- the quality and functionality of the non-infarcted brain tissue. A future nuanced perspective that incorporates handedness or better dominant brain side could significantly enhance patient selection and outcomes in EVT. The paper underscores the complexity of optimizing treatment strategies for stroke patients and highlight areas for future research, such as refining or redefining imaging selection criteria to optimize outcomes for patients with large ischemic cores.

Response: Thank you for your comments. The points were well-taken. We now have added “initial CT perfusion in the early hours usually overestimates the final infarct core. Relying on the CT perfusion mismatch concept to select patients for EVT will inadvertently exclude individuals who could still benefit from reperfusion” and “Finally, it is crucial to move beyond just the quantity of infarcted tissue and consider the infarct's location and eloquent areas- the quality and functionality of the non-infarcted brain tissue. A future nuanced perspective that incorporates handedness or better dominant brain side could significantly enhance patient selection and outcomes in EVT. The current paper underscores the complexity of optimizing treatment strategies for stroke patients and highlights areas for future research, such as refining or redefining imaging selection criteria to optimize outcomes for patients with large ischemic cores.” to the 3^rd^ paragraph of the Discussion section.

General editorial requests:
(Note: not all will apply to your paper, but please check each item carefully)

*       We ask every co-author listed on the manuscript to fill in a contributing author statement, making sure to declare all competing interests. If any of the co-authors have not filled in the statement, we will remind them to do so when the paper is revised. If all statements are not completed in a timely fashion this could hold up the re-review process. If new competing interests are declared later in the revision process, this may also hold up the submission. Should there be a problem getting one of your co-authors to fill in a statement we will be in contact. Please do not add or remove authors without first discussing this with the handling editor. You can see our competing interests policy here: <http://journals.plos.org/plosmedicine/s/competing-interests>.

Response: Done. 

*       Please upload any figures associated with your paper as individual TIF or EPS files with 300dpi resolution at resubmission; please read our figure guidelines for more information on our requirements: <http://journals.plos.org/plosmedicine/s/figures>. While revising your submission, please upload your figure files to the PACE digital diagnostic tool, <https://pacev2.apexcovantage.com/>. PACE helps ensure that figures meet PLOS requirements. To use PACE, you must first register as a user. Then, login and navigate to the UPLOAD tab, where you will find detailed instructions on how to use the tool. If you encounter any issues or have any questions when using PACE, please email us at [PLOSMedicine@plos.org](mailto:PLOSMedicine@plos.org).

Response: Done. 

*       Please ensure that the paper adheres to the PLOS Data Availability Policy (see <http://journals.plos.org/plosmedicine/s/data-availability>), which requires that all data underlying the study's findings be provided in a repository or as Supporting Information. For data residing with a third party, authors are required to provide instructions with contact information (web or email address) for obtaining the data. Please note that a study author cannot be the contact person for the data. PLOS journals do not allow statements supported by "data not shown" or "unpublished results." For such statements, authors must provide supporting data or cite public sources that include it.

Response: Done. 

*       We expect all researchers with submissions to PLOS in which author-generated code underpins the findings in the manuscript to make all author-generated code available without restrictions upon publication of the work. In cases where code is central to the manuscript, we may require the code to be made available as a condition of publication. Authors are responsible for ensuring that the code is reusable and well documented. Please make any custom code available, either as part of your data deposition or as a supplementary file. Please add a sentence to your data availability statement regarding any code used in the study, e.g. "The code used in the analysis is available from Github [URL] and archived in Zenodo [DOI link]" Please review our guidelines at <https://journals.plos.org/plosmedicine/s/materials-software-and-code-sharing> and ensure that your code is shared in a way that follows best practice and facilitates reproducibility and reuse. Because Github depositions can
be readily changed or deleted, we encourage you to make a permanent DOI'd copy (e.g. in Zenodo) and provide the URL.

Response: we have upload data as S1 Data and S2 Data. If further prcedure is needed, we need your assist on it.   

*       FINANCIAL DISCLOSURES: The funding statement should include: specific grant numbers, initials of authors who received each award, URLs to sponsors’ websites. Also, please state whether any sponsors or funders (other than the named authors) played any role in study design, data collection and analysis, the decision to publish, or preparation of the manuscript. If they had no role in the research, include this sentence: “The funders had no role in study design, data collection and analysis, decision to publish, or preparation of the manuscript.”

Response: Done. 

*       COMPETING INTEREST: All authors must declare their relevant competing interests per the PLOS policy, which can be seen here: <https://journals.plos.org/plosmedicine/s/competing-interests>
For authors with ties to industry, please indicate whether any of the interests has a financial stake in the results of the current study.

Response: Done. 

FORMATTING - GENERAL
*       Abstract: Please structure your abstract using the PLOS Medicine headings (Background, Methods and Findings, Conclusions). Please combine the Methods and Findings sections into one section.

Response: Done. 

*       At this stage, we ask that you include a short, non-technical Author Summary of your research to make findings accessible to a wide audience that includes both scientists and non-scientists. The Author Summary should immediately follow the Abstract in your revised manuscript. This text is subject to editorial change and should be distinct from the scientific abstract. Ideally each sub-heading should contain 2-3 single sentence, concise bullet points containing the most salient points from your study. In the final bullet point of 'What Do These Findings Mean?', please include the main limitations of the study in non-technical language. Please see our author guidelines for more information: <https://journals.plos.org/plosmedicine/s/revising-your-manuscript#loc-author-summary>.

Response: Done. 

*       Please express the main results with 95% CIs as well as p values. When reporting p values please report as p<0.001 and where higher as the exact p value p=0.002, for example. Throughout, suggest reporting statistical information as follows to improve clarity for the reader "22% (95% CI [13%,28%]; p</=)". Please be sure to define all numerical values at first use.

Response: Done.

*       Please include page numbers and line numbers in the manuscript file. Use continuous line numbers (do not restart the numbering on each page).

Response: Done.   

*       Please cite the reference numbers in square brackets. Citations should precede punctuation.

Response: Done.

FIGURES AND TABLES
*       Please provide titles and legends for all figures and tables (including those in Supporting Information files).

Response: Done. 

*       Please define all abbreviations used in each figure/table (including those in Supporting Information files).

Response: Done.

*       Please consider avoiding the use of red and green in order to make your figure more accessible to those with color blindness.

Response: The sofware, Revman, use red in certain area and we are unable to change its color. 

SUPPLEMENTARY MATERIAL
*       Please note that supplementary material will be posted as supplied by the authors. Therefore, please amend it according to the relevant comments outlined here

Response: Done. 

*       Please cite your Supporting Information as outlined here: <https://journals.plos.org/plosmedicine/s/supporting-information>

Response: Done. 

REFERENCES
*       PLOS uses the numbered citation (citation-sequence) method and first six authors, et al.

Response: Done. 

*       Please ensure that journal name abbreviations match those found in the National Center for Biotechnology Information (NCBI) databases (<http://www.ncbi.nlm.nih.gov/nlmcatalog/journals>), and are appropriately formatted and capitalised.

Response: Done. 

*       Where website addresses are cited, please include the complete URL and specify the date of access (e.g. [accessed: 12/06/2024]).

Response: Done. 

*       Please also see <https://journals.plos.org/plosmedicine/s/submission-guidelines#loc-references> for further details on reference formatting.

Response: Done. 

STUDY TYPE-SPECIFIC REQUESTS

*       Please report your SR/MA according to the PRISMA guidelines provided at the EQUATOR site. <http://www.equator-network.org/reporting-guidelines/prisma/>. Please provide the completed PRISMA checklist as Supporting Information. When completing the checklist, please use section and paragraph numbers, rather than page numbers. Please add the following statement, or similar, to the Methods: "This study is reported as per the Preferred Reporting Items for Systematic Reviews and Meta-Analyses (PRISMA) guideline (S1 Checklist)."

Response: Done.   

*       Abstract: Please report your abstract according to PRISMA for abstracts (<https://doi.org/10.1371/journal.pmed.1001419>) following the PLOS Medicine abstract structure (Background, Methods and Findings, Conclusions). Please ensure you provide dates of search, data sources, number of studies included, types of study designs included, eligibility criteria, and synthesis/appraisal methods.
Response: Done.

*       Please note that we expect searches to be updated to within 6 months of the time of submission.
Response: Done.
__________________________________________________
In compliance with data protection regulations, you may request that we remove your personal registration details at any time.  (Use the following URL: <https://www.editorialmanager.com/pmedicine/login.asp?a=r>). Please contact the publication office if you have any questions.

Response: We do not have such request.
