# Supplementary material for: Endovascular thrombectomy in acute stroke with a large ischemic core: A systematic review and meta-analysis of randomized controlled trials
Source: PLoS Med. 2025 Apr 17;22(4):e1004484. doi: 10.1371/journal.pmed.1004484 (PMC12037071; doi:10.1371/journal.pmed.1004484)
Supplement: Attachment — Submitted filename: Respond to Comments from Editor.docx [file pmed.1004484.s018.docx]

Comments from Reviewers:

Reviewer #2: The authors have addressed my points

Michael Dewey

Any attachments provided with reviews can be seen via the following link:

------------------------------------------------------------

Requests from Editors:

GENERAL

1. * Please ensure that your manuscript contains line and page numbers.

Response: Thank you for your comments. We now have ensured that our manuscript contains line and page numbers.

2. * Please confirm that your title complies with PLOS Medicine's style. Your title must be nondeclarative and not a question. It should begin with main concept if possible. "Effect of" should be used only if causality can be inferred, i.e., for an RCT. Please place the study design ("A randomized controlled trial," "A retrospective study," "A modelling study," etc.) in the subtitle (ie, after a colon).

Response: Thank you for your comments. We now have revised it as “Association between Endovascular Thrombectomy and Outcomes in Acute Stroke with a Large Ischemic Core: A Systematic Review and Meta-analysis of Randomized Controlled Trials”.

3. * Your study is observational and therefore causality cannot be inferred. Please remove language that implies causality, such as effect. Refer to associations instead.

Response: Thank you for your comments. We now have revised the title as “Association between Endovascular Thrombectomy and Outcomes in Acute Stroke with a Large Ischemic Core: A Systematic Review and Meta-analysis of Randomized Controlled Trials”.

4. * Please ensure that all abbreviations are defined at first use throughout the text (including statistical abbreviations). Please also check figures and tables.

Response: Thank you for your comments. We now have ensured that all abbreviations are defined at first use throughout the text, figures and tables.

5. * Please review your text for claims of novelty or primacy (e.g. 'for the first time', ‘novel’) and remove this language.

Response: Thank you for your comments. We now have reviewed our text and ensured this language were not used.

6. * Please check that any use of statistical terms (such as trend or significant) are supported by the data, and if not please remove them.

Response: Thank you for your comments. We have checked that all statistical terms are supported by the data.

7. * Statistical reporting: Please revise throughout the manuscript, including tables and figures.

a) Please report statistical information as follows to improve clarity for the reader "22% (95% CI [13%,28%]; p</=)".

Response: Thank you for your comments. We have done this.

b) Please separate upper and lower bounds with commas instead of hyphens as the latter can be confused with reporting of negative values.

Response: Thank you for your comments. We have done this.

c) Please define statistical definitions at first use and repeat the abbreviated definitions (HR, CI etc.) for each set of parentheses.

Response: Thank you for your comments. We have done this.

8. * Please revise for use of patient-centered language. Please note that patient-centered language is constructed with the use of post-modified nouns (e.g. 'patients with ischemia’ (or similar) instead of ‘ischemic patients’) putting the person first in the sentence structure.

Response: Thank you for your comments. We now have used patient-centered language (e.g. patients with ischemic stroke) throughout the manuscript.

9. * The terms gender and sex are not interchangeable (as discussed in https://www.who.int/health-topics/gender#tab=tab_1 ); please use the appropriate term. Since you are referring to sex, please use the terms ‘females/males’ instead of ‘women/men’ in your study.

Response: Thank you for your comments. We now have used use the terms ‘females/males’ instead of ‘women/men’ in our study.

10. * p.35: Please ensure that the information provided here matches the metadata provided in the online submission form.

Response: Thank you for your comments. We have ensured that the information provided here matches the metadata provided in the online submission form.

11. * Throughout the manuscript you refer to 'at 90 days' without specifying what this time point refers to (e.g. 90 days post-intervention). Please clarify and revise throughout.

Response: Thank you for your comments. We now have revised the description as “at 90 days post-stroke” throughout the manuscript.

12. * Please clarify throughout the manuscript that you are comparing endovascular thrombectomy plus medical care to medical care alone (you often write endovascular thrombectomy compared to medical care alone).

Response: Thank you for your comments. We now have revised the description as “endovascular thrombectomy plus medical care compared to medical care alone” throughout the manuscript.

13. * Please consider if moving some of the figures shared in the supplementary information into the main text would aid the reader.

Response: Thank you for your comments. We now have moved some of the figures shared in the supplementary information into the main text would aid the reader.

DATA AVAILABILITY

14. * Please clarify what you mean by “we upload the data for outcome calculation”. Please clarify what type of data you are referring to and where the data will be uploaded (e.g. Supporting Information, repository etc.).

Response: Thank you for your comment. In accordance with the journal’s technical requirements, we have revised the data availability statement to:

“All relevant data are within the manuscript and its Supporting Information files.”

ABSTRACT

15. * Please confirm that your abstract complies with our requirements, including providing all the information relevant to this study type https://journals.plos.org/plosmedicine/s/submission-guidelines#loc-abstract

Response: Confirmed.

16. * In the last sentence of the Abstract Methods and Findings section, please describe the main limitation(s) of the study's methodology.

Response: Thank you for your comments. We have added “Main limitations include variability in imaging definitions of large core and inclusion of both larger moderate and true large cores in the analysis.” in the last sentence of the Abstract Methods and Findings section.

17. * Please ensure that all numbers presented in the abstract are present and identical to numbers presented in the main manuscript text.

Response: Confirmed.

18. * Please mention whether there were any language restriction for the search.

Response: Thank you for your comments. We have added “There were no language restrictions.” to the Abstract Methods and Findings section.

19. * Please provide the synthesis/appraisal methods.

Response: Thank you for your comments. We have added “We computed the random-effects estimate based on the inverse variance method when 2 or more studies provided sufficient data for a given outcome.” and “Grading of Recommendations Assessment, Development and Evaluations (GRADE) was used to evaluate summaries of evidence for the outcomes.” to the Abstract Methods and Findings section.

20. * Please clarify why for the outcome on reduced disability you have provided minimum and maximum possible NNT (instead of 95% CI values).

Response: We are grateful for this query. The NNT value for improving by 1 or more levels on the mRS was derived from the treatment group mRS distributions by applying the min-max algorithmic method. In this method, the first step is to derive algorithmically the two most extreme possible NNT values compatible with the observed mRS distributions. At one extreme, the joint outcome table is algorithmically completed under the assumption that every patient who benefitted improved by the least number of mRS levels compatible with the observed final distributions. In this scenario, many patients benefit a little. Accordingly, since the most possible patients benefit, the NNT is the lowest possible value. At the other extreme, the joint outcome table is algorithmically completed under the assumption that every patient who benefitted improved by the highest number of mRS levels compatible with the observed final distributions. In this scenario, fewer patients benefit, but each patient benefits a lot. Accordingly, since the fewest possible patients benefit, the NNT is the highest possible value. The final NNT estimate is then derived by taking the average of the lowest and highest possible values. With this approach, the NNT estimated (the average NNT value) and range (the two extreme values) can be derived and reported, as in the manuscript. This technique does not permit derivation of 95% confidence intervals around the estimated. Due to word constraints, we omitted this from the Abstract but retained it in the main text.

21. * The term trend should be used only when the test for trend has been conducted. Please revise accordingly.

Response: Thank you for your comments. We now have revised the relevant statement in Abstract, and other parts of the manuscript, as “EVT plus medical care, compared with medical care alone, was associated with a nonsignificantly higher proportion of patients with early symptomatic intracranial hemorrhage”.

AUTHOR SUMMARY

22. * Please revise the format and insert bullet points.

Response: Thank you for your comments. We have revised the format and insert bullet points.

23. * Please clarify that you compared endovascular thrombectomy plus medical care to medical care alone.

Response: Thank you for your comments. We have clarified that we compared endovascular thrombectomy plus medical care to medical care alone.

24. * Please clarify that the results refer to 90 days post-procedure.

Response: Thank you for your comments. We have revised the statement as 90 days post-stroke.

INTRODUCTION

25. * Given that PLOS Medicine has a wide readership, we suggest that the introduction could briefly explain what endovascular thrombectomy is, but we leave you to decide.

Response: Thank you for your comments. We now have added “EVT is a minimally invasive procedure in which a catheter is inserted into a large artery, typically in the groin, and advanced to the blocked brain artery to remove the clot, restoring blood flow.” to the Introduction.

METHODS AND RESULTS

26. * Please include a flow diagram as Figure 1 (according to the PRISMA guidelines).

Response: Thank you for your comments. We now have included a flow diagram as Figure 1.

27. * Please briefly mention what the modified Rankin scale measures, e.g. that it is a 6-level ordinal categorical scale outcome measure used primarily to classify the degree of disability in stroke patients.

Response: Thank you for your comments. We have added a brief explanation in the Methods section, stating that the modified Rankin Scale (mRS) is a 7-point ordinal scale (ranging from 0 to 6) commonly used to classify the degree of disability among patients who have experienced a stroke.

28. * p.8: Do the search terms you present on page 8 reflect the full search strategy?

Response: Thank you for your comments. Yes, the search terms presented on page 8 reflect the full search strategy used in our literature search.

29. * p.12: Please explain the Grading of Recommendations in more detail.

Response: Thank you for your comment. We have revised the text to:

“Grading of Recommendations Assessment, Development and Evaluation (GRADE) was used to assess the certainty of evidence for each outcome, considering factors such as risk of bias, inconsistency, indirectness, imprecision, and publication bias [11,19].”

30. * p.11: “All statistical tests for heterogeneity are weak, including I².” – please rephrase and avoid making absolute statements based on a single reference. We think it would be better to focus on the fact that I² should be interpreted with caution when a meta-analysis has few studies.

Response: Thank you for your comments. We now have revised the statement as “Given the small number of trials in our study, I² should be interpreted with caution [17]”.

31. * p.14, “73%” – we suggest providing the numerator and denominator for transparency.

Response: Thank you for your comments. We now have revised the statement as “Successful reperfusion, defined as a Thrombolysis in Cerebral Infarction (TICI) score of 2b or 3, was achieved in 107 of 146 patients (73%) in the TESLA trial's EVT group”.

32. * p.14: Please briefly describe the results for the risk of bias assessment.

Response: Thank you for your comment. We have added the following statement to the manuscript:

“All included trials were judged to have a high risk of bias due to deviations from intended interventions, primarily because they were not blinded.”

33. * Table 1:

a) Please define ‘y’ (years) or spell out 'y'.

Response: Thank you for your comments. We now have spelled out “y” as “years”.

b) For the category ‘Age’, please clarify whether you are presenting mean or median.

Response: Thank you for your comments. We now have added “*: RESCUE-Japan LIMIT used mean age while other trials used median age.” in the footnote of Table 1.

c) Please also present ranges (e.g. standard deviation or IQR) when presenting mean or median.

Response: Thank you for your comments. We now have added ranges (e.g. standard deviation or IQR) when presenting mean or median.

34. * Figure 1: Please define ‘IV’ and ‘CI’. Please note that in the axis title it says “Favors Meical Care” – please correct. Please clarify what ‘at 90 days’ mean (at 90 days post ?).

Response: Thank you for your comments. We have moved original Fig 1 to Fig 2 and have corrected these issues accordingly.

Revised Figure 2 Title & Legend:

Fig 2. Title: Functional independence at 90 days post-stroke

Legend:

Risk ratio with 95% confidence interval for functional independence, defined as a modified Rankin Scale (mRS) score of 0–2, comparing EVT plus medical care with medical care alone in acute stroke with a large ischemic core due to ICA or MCA M1 occlusion at 90 days post-stroke.

CI, confidence interval; EVT, endovascular thrombectomy; ICA, internal carotid artery; IV, inverse variance; MC, medical care; MCA, middle cerebral artery; M1, M1 segment of middle cerebral artery.

35. * Figure 2: Please correct ‘Endovascular Thrombecomy’. Please clarify that the first group entails ‘Endovascular Thrombectomy plus medical care’. Please clarify what ‘at 90 days’ mean (at 90 days post ?).

Response: Thank you for your comments. We have moved original Fig 2 to Fig 3. We have revised the first group label to “EVT plus medical care”. Additionally, we have clarified that 'at 90 days' refers to 'at 90 days post-stroke.

Revised Figure 3 Title & Legend:

Fig 3. Title: Distribution of modified Rankin Scale scores at 90 days post-stroke

Legend:

A modified Rankin Scale (mRS) score of 0–1 indicates nondisabled, 2 indicates disabled but independent, 3 indicates dependent but ambulatory, 4 indicates not ambulatory but not requiring constant care, 5 indicates requiring constant care, and 6 indicates death.

EVT, endovascular thrombectomy.

36. * Figure 3: Please define ‘IV’ and ‘CI’. Please clarify what ‘at 90 days’ mean (at 90 days post ?).

Response: Thank you for your comments. We have moved original Fig 3 to Fig 8. We have clarified that 'at 90 days' refers to 'at 90 days post-stroke.

Revised Figure 8 Title & Legend:

Fig 8. Title: Requiring constant care or death at 90 days post-stroke

Legends: Risk ratio with a 95% confidence interval for requiring constant care or death, defined as a modified Rankin Scale (mRS) score of 5–6, comparing EVT plus medical care with medical care alone in acute stroke with a large ischemic core due to ICA or MCA M1 occlusion at 90 days post-stroke.

CI, confidence interval; EVT, endovascular thrombectomy; ICA, internal carotid artery; IV, inverse variance; MC, medical care; MCA, middle cerebral artery; M1, M1 segment of middle cerebral artery.

37. * p.23: “associated with a trend toward higher proportion of patients“ - the term trend should be used only when the test for trend has been conducted. Please revise accordingly.

Response: Thank you for your comments. We appreciate your feedback and have revised the sentence to read: “Pooled results showed that EVT plus medical care, compared with medical care alone, was associated with a nonsignificantly higher proportion of patients with early symptomatic intracranial hemorrhage…”.

38. * p.24: Please describe the results for GRADE in the main text in more detail. We suggest moving the table into the main results.

Response: Thank you for your comment. We have moved the GRADE summary table to Table 2 in the main Results section. We also added the following sentence to the main text:

"The GRADE assessment indicated high-certainty evidence for the outcomes of functional independence, being nondisabled, and early neurologic improvement, and moderate-certainty evidence for improved functional outcomes, ambulation, requiring constant care or death, death, and early symptomatic intracranial hemorrhage."

39. * S8 Figure: Please note that the figure seems to be minimally cut-off at the lower edge.

Response: Thank you for your comment. We have moved S8 Fig to S1 Fig. We have adjusted figure and there is no minimally cut-off at the lower edge.

40. * p.27: Please expand the section on the funnel plot (e.g. mention that the funnel plot is used to detect publication bias) or include the results elsewhere. We do not think it’s necessary that a one liner requires its own section with its own subheading.

Response: Thank you for your comment. We have delete its own subheading and the revised statement is “The funnel plot for the primary outcome did not show major asymmetry (S1 Fig).”

41. * The results section currently has many subheadings, which we suggest should be reduced. Some of the subheadings could instead be used to introduce a paragraph. For example, on page 21, Safety Outcomes: "For the first safety outcome of requiring constant care or death at 90 days (mRS score of 5 to 6), the pooled results showed that EVT...". Please revise throughout accordingly.

Response: Thank you for your comments and we now have removed some of the subheading. We adopted your suggestions and revised throughout accordingly.

DISCUSSION

42. * Please remove any subheadings in the Discussion, including the Conclusion subheading.

Response: Thank you for your comments. We now have removed all subheadings in the Discussion.

43. * p.27 “less disabled outcomes” – please re-phrase and revise accordingly throughout.

Response: Thank you for your comments. We now have rephrased it as “achieving better functional outcomes” throughout.

44. * p.28, please change to: “appears to be evidence-based and clinically justified”.

Response: Thank you for your comments. We now have changed to the statement to “appears to be evidence-based and clinically justified”

45. * p.31: We feel that you jump very abruptly to stating that the current study differs from the study by Chen et al. Please revise and, for example, start the paragraph by stating that a similar meta-analysis has recently been published.

Response: Thank you for your comments. We have added a sentence to introduce the comparison more smoothly:

“A similar meta-analysis was published by Chen et al., which also investigated the efficacy of EVT in patients with acute stroke and a large ischemic core.”

46. * p.31, “we were unable to conduct subgroup analyses” – please clarify since you have conducted a subgroup analysis.

Response: Thank you for your comments. We now have deleted “since this is a study-level meta-analysis, we were unable to conduct subgroup analyses because each trial reported its subgroup analysis based on its original primary outcome. An individual participant pooled analysis of all trials is warranted to solve this problem.”

Journal Requirements:

1) Please ensure that the funders and grant numbers match between the Financial Disclosure field and the Funding Information tab in your submission form. Note that the funders must be provided in the same order in both places as well.

Currently, (Chiayi Chang Gung Memorial Hospital CMRPG6H0191 Meng Lee) is missing from the Financial Disclosure

Response: Thank you for your comments. We have now revised the Financial Disclosure section in the manuscript and the Funding Information tab to state 'None' for consistency.

2) We noticed that you used “data not shown” in the manuscript. We do not allow these references, as the PLOS data access policy requires that all data be either published with the manuscript or made available in a publicly accessible database. Please amend the supplementary material to include the referenced data or remove the references.

Response: Thank you for your comments. We have ensured we did not use “data not shown” in the manuscript.

3) In your Data Availability Statement, please ensure you have provided all necessary access information in complete sentences. Access information should include the name of any data repositories used as well as digital object identifiers (DOIs), accession numbers or codes, or other persistent identifiers for all relevant data. If all data has been provided within your submission files, please use the statement "All relevant data is available within the manuscript and Supporting Information files." The Data Availability Statement will appear on the published version of your manuscript.

Response: Thank you for your comments. We now have used the statement "All relevant data is available within the manuscript and Supporting Information files." as our Data Availability Statement.
