# Supplementary material for: Endovascular thrombectomy in acute stroke with a large ischemic core: A systematic review and meta-analysis of randomized controlled trials
Source: PLoS Med. 2025 Apr 17;22(4):e1004484. doi: 10.1371/journal.pmed.1004484 (PMC12037071; doi:10.1371/journal.pmed.1004484)
Supplement: Attachment — Submitted filename: 3rd response to Requests from Editors.docx [file pmed.1004484.s019.docx]

Requests from Editors:

1) Title: We suggest changing the title to: Endovascular Thrombectomy in Acute Stroke with a Large Ischemic Core: A Systematic Review and Meta-analysis of Randomized Controlled Trials

Response: Thank you for your comments. We have changed the title to: Endovascular Thrombectomy in Acute Stroke with a Large Ischemic Core: A Systematic Review and Meta-analysis of Randomized Controlled Trials.

2) Abstract: Please change the search dates to “January 1 2000 to September 25 2024”.

Response: Thank you for your comments. We have changed the search dates to “January 1 2000 to September 25 2024”. in Abstract

3)Abstract: Please note that we have some flexibility on the length of the abstract, i.e. you may include the NNT number (plus minimum and maximum NNT) for functional outcomes/reduced disability, and we encourage you to do so.

Response: Thank you for your comments. We have included (NNT, 4 [minimum possible NNT, 2; maximum possible NNT, 6]) for reduced disability.

4) Abstract: Given that you have described the primary secondary outcome as reduced disability, we feel that the description of "better functional outcomes" later on in the Abstract may be confusing to readers. Please revise and use the same description throughout the manuscript. You seem to have changed "reduced disability" to "better functional outcomes" throughout, so we would prefer to stick with the latter.

Response: Thank you for your reminder. We carefully consider between these two terms and decided to use “reduced disability” throughout, in line with the term used in the landmark EVT paper of Goyal M. Lancet 2016 (ref 1 in our paper) and Stroke expert consensus (ref 15 in our paper),

5) Introduction: Thank for including a brief introduction on endovascular thrombectomy. Please provide a reference.

Response: Thank you for your comments. We have provided a reference [1].

6) Figure 1: In the second box on the right, please change the text to “Records excluded due to duplicate”.

Response: Thank you for your comments. We have change the text to “Records excluded due to duplicate” in the second box on the right of Fig 1.

7) Table 2: Please define ‘mRS’ and replace commas with hyphens in the effect column.

Response: Thank you for your comments. We have added “mRS, modified Rankin Scale;” and replace commas with hyphens in the effect column.

8) Please note that there are still instances in tables (e.g. Table 4) where you report values, e.g. confidence intervals, with commas instead of hyphens. Please carefully check and revise throughout.

Response: Thank you for your comments. We have carefully checked all tables to ensure we used commas instead of hyphens.

9) Please ensure to upload the completed PRISMA checklist.

Response: Thank you for your comments. We have ensured we uploaded the completed PRISMA checklist.

10) Table 1: Please change "men±SD" to "mean±SD" (fourth row, age).

Response: Thank you for your reminder. We have corrected it.

Journal Requirements:

1) We have noticed that you have a list of Supporting Information legends " S1 Checklist, S1 Table, S2 Table," in your manuscript. However, there are no corresponding files uploaded to the submission. Please upload them as separate files with the item type 'Supporting Information'.

Response: Thank you for your reminder. We have ensured we uploaded S1 Checklist, S1 Table, S2 Table.
